# Supplementary material for: Role of Agricultural Management in the Provision of Ecosystem Services in Warm Climate Vineyards: Functional Prediction of Genes Involved in Nutrient Cycling and Carbon Sequestration
Source: Plants (Basel). 2023 Jan 23;12(3):527. doi: 10.3390/plants12030527 (PMC9919410; doi:10.3390/plants12030527)
Supplement: Supplementary file 1 [file plants-12-00527-s001.zip › plants-2144879-supplementary/Table S1.pdf]

	O1		O2		O3		O4	
	**Mean**	**SE**	**Mean**	**SE**	**Mean**	**SE**	**Mean**	**SE**
Plant cover	20.0275	5.8573	14.4350	6.8717	56.9400	18.2955	57.5600	7.4933
Species richness	4.0000	1.2247	7.0000	1.0801	10.5000	2.0207	7.0000	1.4720
Diversity	0.0759	0.0690	0.0222	0.0071	0.5291	0.1090	0.0049	0.0016
Species								
Anacyclus clavatus/Anthemis arvensis	-	-	-	-	0.0050	0.0029	0.0025	0.0025
Anagallis arvensis	-	-	0.0050	0.0029	-	-	0.0025	0.0025
cf. Arabidopsis thaliana	-	-	0.0025	0.0025	-	-	-	-
Asteraceae	0.0025	0.0025	0.0050	0.0029	**9.3775**	**3.5882**	0.0050	0.0029
Calendula arvensis	-	-	-	-	**1.8800**	**1.8733**	-	-
Cerastium sp.	-	-	-	-	-	-	0.0025	0.0025
Chenopodiaceae	-	-	-	-	0.0050	0.0029	-	-
Convolvulus arvensis	-	-	0.0025	0.0025	0.0025	0.0025	-	-
Diplotaxis sp.	**18.1250**	**5.8962**	**14.3750**	**6.8750**	0.0050	0.0029	**57.5000**	**7.5000**
Dittrichia viscosa	0.0025	0.0025	-	-	-	-	-	-
Echium sp.	-	-	-	-	-	-	0.0075	0.0025
Erodium chium	-	-	0.0075	0.0025	**1.8800**	**1.8733**	0.0025	0.0025
Erodium sp. 1 (type cicutarium)	-	-	0.0075	0.0025	-	-	-	-
Erodium sp. 2 (type moschatum)	0.0025	0.0025	0.0025	0.0025	**12.5025**	**8.2903**	-	-
Filago sp.	-	-	-	-	0.0075	0.0025	-	-
Galium sp.	-	-	0.0100	-	0.0050	0.0029	0.0050	0.0029
Geranium molle	**1.8800**	**1.8733**	**-**	**-**	**16.2525**	**7.1788**	0.0075	0.0025
Lamium amplexicaule	0.0025	0.0025	-	-	-	-	-	-
Leontodon sp.	**-**	**-**	**-**	**-**	**3.7525**	**3.7492**	**-**	**-**
Malva sp.	0.0025	0.0025	0.0025	0.0025	0.0025	0.0025	0.0025	0.0025

Medicago sp.	0.0025	0.0025	-	-	0.0025	0.0025	0.0100	-
Papaver sp.	-	-	0.0050	0.0029	-	-	-	-
Poaceae	-	-	0.0050	0.0029	**7.5025**	**3.0598**	0.0025	0.0025
Senecio sp.	-	-	-	-	0.0025	0.0025	-	-
Silene sp.	-	-	-	-	0.0025	0.0025	-	-
Stellaria sp.	0.0025	0.0025	0.0025	0.0025	-	-	-	-
Taraxacum sp.	**-**	**-**	**-**	**-**	**3.7525**	**2.1636**	**-**	**-**
Urtica urens	-	-	-	-	-	-	0.0075	0.0025
Veronica sp.	0.0050	0.0029	-	-	-	-	0.0025	0.0025
Unidentifiable	-	-	0.0025	0.0025	-	-	-	-
Plant family								
Asteraceae	0.0050	0.0029	0.0050	0.0029	**18.7775**	**7.1872**	0.0075	0.0048
Boraginaceae	-	-	-	-	-	-	0.0075	0.0025
Brassicaceae	**18.1250**	**5.8962**	**14.3775**	**6.8775**	0.0050	0.0029	**57.5000**	**7.5000**
Caryophyllaceae	0.0025	0.0025	0.0025	0.0025	0.0025	0.0025	0.0025	0.0025
Chenopodiaceae	-	-	-	-	0.0050	0.0029	-	-
Convolvulaceae	-	-	0.0025	0.0025	0.0025	0.0025	-	-
Fabaceae	0.0025	0.0025	-	-	0.0025	0.0025	0.0100	-
Geraniaceae	**1.8825**	**1.8725**	0.0175	0.0048	**30.6350**	**15.2147**	0.0100	0.0041
Lamiaceae	0.0025	0.0025	-	-	-	-	-	-
Malvaceae	0.0025	0.0025	0.0025	0.0025	0.0025	0.0025	0.0025	0.0025
Papaveraceae	-	-	0.0050	0.0029	-	-	-	-
Poaceae	**-**	**-**	0.0050	0.0029	**7.5025**	**3.0598**	0.0025	0.0025
Primulaceae	-	-	0.0050	0.0029	-	-	0.0025	0.0025
Rubiaceae	-	-	0.0100	-	0.0050	0.0029	0.0050	0.0029
Urticaceae	-	-	-	-	-	-	0.0075	0.0025

Veronicaceae	0.0050	0.0029	-	-	-	-	0.0025	0.0025
Unidentifiable	-	-	0.0025	0.0025	-	-	-	-
